# Selection of reference genes for expression analysis of plant-derived microRNAs in *Plutella xylostella* using qRT-PCR and ddPCR

**DOI:** 10.1371/journal.pone.0220475

**Published:** 2019-08-01

**Authors:** Lingling Zhang, Xiaodong Jing, Wei Chen, Jianlin Bai, Liette Vasseur, Weiyi He, Minsheng You

**Affiliations:** 1 State Key Laboratory for Ecological Pest Control of Fujian and Taiwan Crops, Institute of Applied Ecology, Fujian Agriculture and Forestry University, Fuzhou, China; 2 International Joint Research Laboratory of Ecological Pest Control, Ministry of Education, Fujian Agriculture and Forestry University, Fuzhou, China; 3 Department of Biological Sciences, Brock University, St. Catharines, Ontario, Canada; Chinese Academy of Agricultural Sciences Institute of Plant Protection, CHINA

## Abstract

The establishment of an expression quantification system that can be easily applied for the comparison of microRNAs (miRNAs) from biological samples is an important step toward understanding functional mechanisms in organisms. However, there is lack of attention on the selection of reference genes for miRNA expression profiling in insect herbivores. Here, we explored the candidate reference genes in a notorious pest of cruciferous crops, *Plutella xylostella*, for normalization of miRNA expression in developmental stages and tissues and in response to a change of food source from artificial diet to host plant *Arabidopsis thaliana*. We first compared the expression levels and stability of eight small RNAs using qRT-PCR, and found that miR11 was the most suitable reference gene for expression quantification of the miRNAs. We then confirmed this finding using digital droplet PCR and further validated with a well-studied cross-kingdom miRNA derived from *A*. *thaliana* (ath-miR159a). However, none of the reference genes was applicable for all experimental conditions, and multiple reference genes were sometimes required within the same experiment. Our work provides a method for the selection of reference genes for quantification of plant-derived miRNAs, which paves the way for unveiling their roles in the insect-plant coevolution.

## Introduction

MicroRNAs (miRNAs) are a class of single-stranded noncoding RNAs characterized by their short length ranging from 18 to 24 nucleotides and universally exist across various living organisms [1]. MiRNAs function at the posttranscriptional level by silencing gene expression or protein translation and play pivotal roles in many biological processes in insects, such as cell growth, immune response, and apoptosis [2]. Establishing a quantitative expression profiling platform for the comparison of miRNAs from biological samples is the basis for further functional studies.

Fluorescence-based quantitative real time reverse transcriptase PCR (qRT-PCR) is widely used for relative quantification of gene expression [3, 4]. A proper reference gene is required for the normalization of gene expression in qRT-PCR experiments to ensure results are comparable among the tested samples [5, 6]. This method is also applicable for the quantification of miRNA expression, in which the U6 snRNA (U6) or 5S rRNA is commonly used as the reference gene. However, the expression of these two small RNAs may become unstable under some specific conditions [7, 8]. Further, under the various biotic and abiotic stresses, some other reference genes have been found suitable for the quantification of corresponding miRNA expressions [9–11]. Based on screening of high-throughput sequencing data of the insect pest *Helicoverpa armigera*, eight miRNAs and two ribosomal RNAs with constitutive expression patterns were selected as candidate reference genes to normalize qRT-PCR data, and no single reference gene was suitable for all experimental conditions [12].

In contrast to qRT-PCR, the droplet digital PCR (ddPCR) is used for the absolute quantification of gene expression, in which the PCR is performed in discrete and volumetrically defined water-in-oil droplet partitions, providing more precise and sensitive results and a wider detection range [13–15]. Sample partitioning mitigates the effects of target competition, making PCR amplification less sensitive to inhibition and greatly improving the discriminatory capacity of assays [16]. Importantly, the quantification of gene expression using ddPCR is independent of the reference gene and standard curves [17] and is especially suitable for measuring low-abundance miRNAs [18, 19]. There are two detection methods for ddPCR based on EvaGreen dye and TaqMan probe. Studies have shown that these two methods are equally reliable in circulating miRNA quantification in human plasma and blood [20]. However, ddPCR is relatively costly and time-consuming, which prevents its wider application compared with qRT-PCR.

The diamondback moth (*Plutella xylostella*) is a worldwide notorious specialist insect attacking cruciferous crops [21]. It has the ability to detoxify the glucosinolates of host plants through a specific sulfatase [22, 23]. Given its capacity to rapidly develop resistance to most classes of insecticide, biologically-based approaches for its control are increasingly needed [24, 25]. A better understanding of the function of the miRNA-centered gene expression regulation system in *P*. *xylostella* may provide an alternative approach for the management of this insect pest. However, information on the selection of suitable reference genes for miRNA expression quantification in *P*. *xylostella* remains limited [26]. In this study, we tested eight reference genes including seven miRNAs and one ribosomal RNA of *P*. *xylostella* from small RNA sequencing data for their application in the qRT-PCR-based miRNA expression quantification. Using this system, we successfully detected the well-studied plant-derived miR159a, and these results were further validated using ddPCR. The work provided a method for identifying optimal reference genes for miRNA expression quantification in *P*. *xylostella* and paved a way for unveiling the roles of plant-derived miRNAs in cross-kingdom gene expression regulation.

## Results

### Expression levels of candidate reference genes

**Fig 1:**
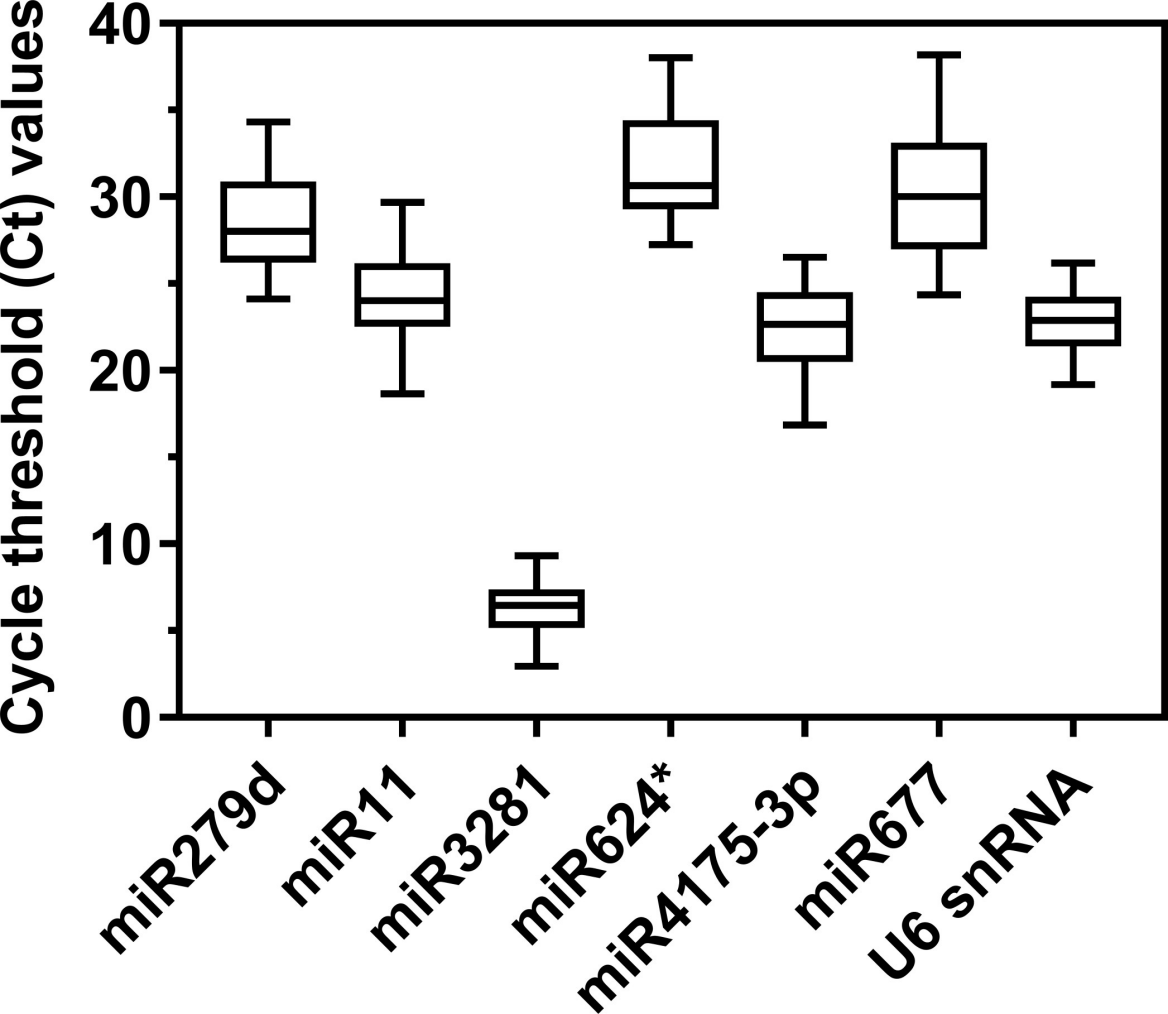
Cycle threshold (Ct) values of candidate reference genes. The Ct values of the candidate reference genes were measured in the samples from G88 and GC strains as described in the Materials and Methods.

Results showed that six candidate reference genes displayed PCR efficiency (*E*) values ranging from 94.7% to 109.6% with a correlation coefficient (R^2^) > 0.98 (Table 1 and S1 Fig). miR2b-5p and miR677 with abnormal PCR efficiencies were excluded for further analysis. There was no nonspecific amplification in qRT-PCR (S2 Fig). Among the tested samples, most candidate miRNAs exhibited a moderate expression levels with Ct values greater than 15 cycles with the exception of miR3281, which produced a Ct value less than 10 cycles (Fig 1). We therefore excluded miR3281 for further analysis to avoid deviation in the expression levels of reference genes. In all samples, U6 displayed the lowest dispersion while miR624* showed the highest variation (Fig 1 and S1 Table). Finally, four miRNAs, miR279d, miR11, miR624*, and miR4175-3p, and U6 were used for stability analysis.

**Table 1:** Evaluation of primers used for qRT-PCR.

Name	Sequence (5'-3')	E (%)	R^2^	Ta (°C)
miR-279d	RT: CTCAACTGGTGTCGTGGAGTCGGCAATTCAGTTGAGAGGATGAG	109.6%	0.992	58
	F: ACACTCCAGCTGGGTGACTAGATTTTCA			
miR-11	RT: CTCAACTGGTGTCGTGGAGTCGGCAATTCAGTTGAGAGCTAGAA	100.3%	0.986	58
	F: ACACTCCAGCTGGGCATCACAGTCAGAG			
miR-3281	RT: CTCAACTGGTGTCGTGGAGTCGGCAATTCAGTTGAGACACGCCA	94.7%	0.983	58
	F: ACACTCCAGCTGGGAGAAATCTTATGTCGATG			
miR-624*	RT: CTCAACTGGTGTCGTGGAGTCGGCAATTCAGTTGAGTGAGACTA	108.8%	0.995	58
	F: ACACTCCAGCTGGGTATTCACCAGTACTTG			
miR-4175-3p	RT: CTCAACTGGTGTCGTGGAGTCGGCAATTCAGTTGAGTCTACCAT	108.4%	0.996	58
	F: ACACTCCAGCTGGGGGCGTAGCTCAG			
U6	F: CTCGCTTCGGCAGCACA	108.4%	0.996	58
	RT&R: AACGCTTCACGAATTTGCGT			
Universal	R: TGGTGTCGTGGAGTCG	/	/	58

RT, reverse transcription primer; F, forward primer; R, reverse primer; E, PCR efficiency; R^2^, regression coefficient; Ta, annealing temperature. The sequence underlined in forward primer denotes the reverse complementary sequence of the last 8 bp of mature miRNA, and the sequence underlined in reverse transcription primer represents the remainder sequence of mature miRNA.

### Stability of candidate reference genes in developmental stages and tissues of G88 strain

**Fig 2:**
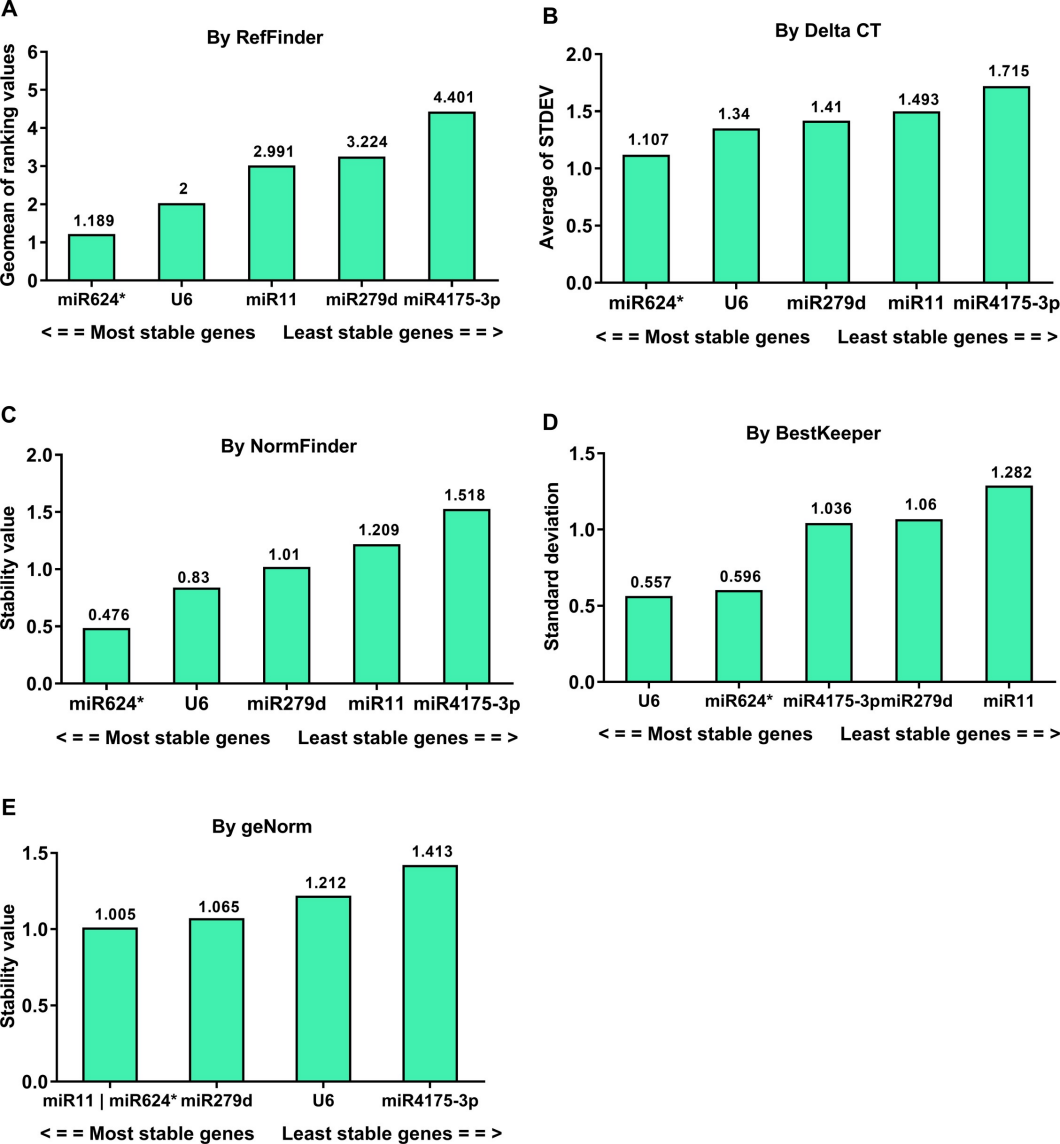
Stability analysis of candidate reference genes in the developmental stages of G88 strain. The Ct values derived from samples including first-day eggs, 1^st^ instar larvae, 2^nd^ instar larvae, 3^rd^ instar larvae, 4^th^ instar larvae, pupae and adults were used to calculate the stability values by different algorithms. A lower value indicates a more stable candidate gene as reference gene (numbers reported above the columns).

**Fig 3:**
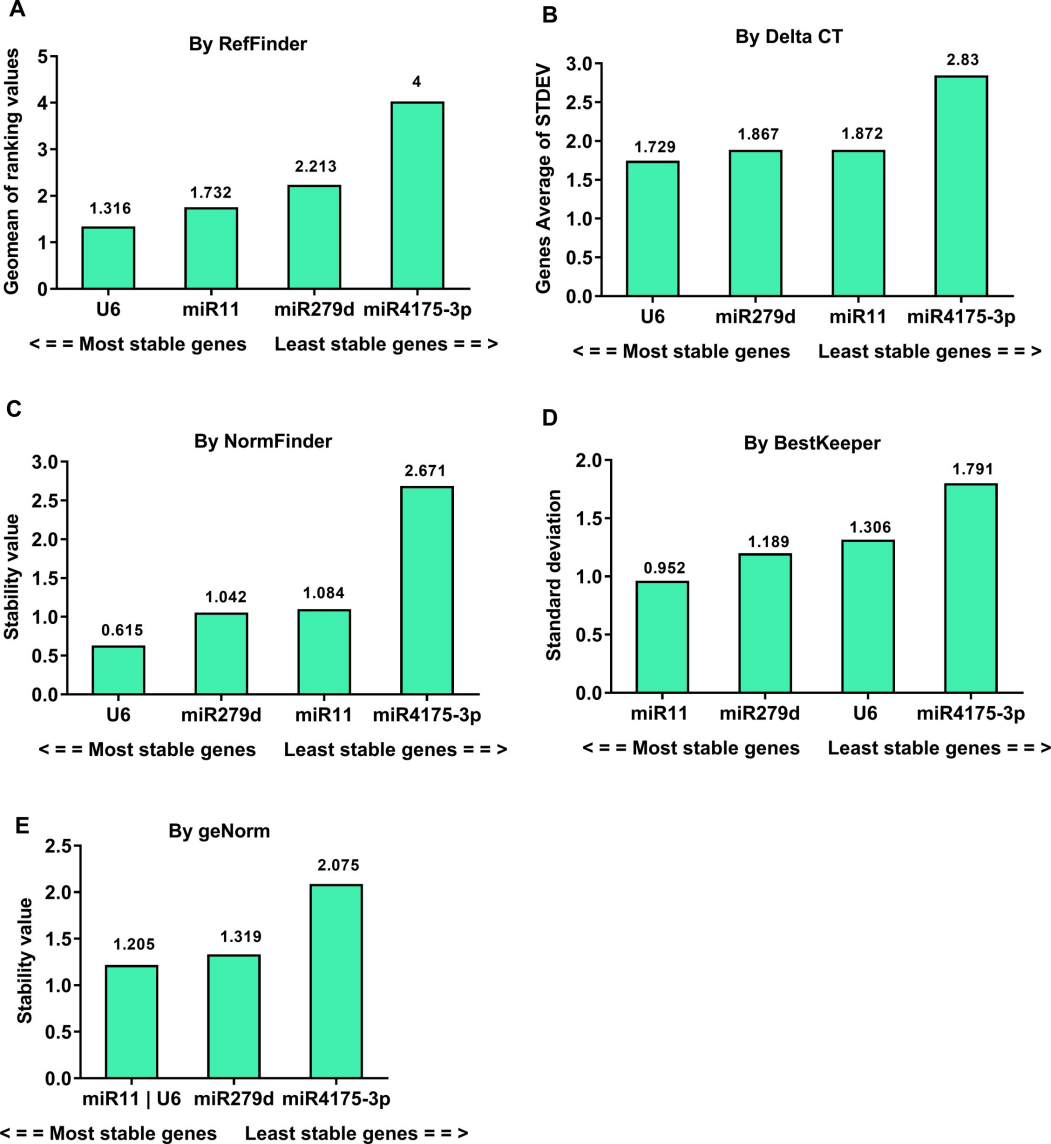
Stability analysis of candidate reference genes in the tissues of G88 strain. The Ct values derived from samples including brain, midgut, silk gland, Malpighian tubule, fat body, hemolymph, and the remaining tissues of 4^th^ instar larvae were used to calculate the stability values by different algorithms. A lower value indicates a more stable candidate gene as reference gene (numbers reported above the columns).

The comprehensive ranking calculated by RefFinder identified miR624* as the most stable reference gene over all the developmental stages (Fig 2A). Rankings varied with Delta CT and NormFinder identifying miR624*, miR279d, and U6 as the most stable genes among developmental stages (Fig 2B and 2C). In contrast, BestKeeper selected U6, miR624*, and miR4175-3p (Fig 2D), and geNorm ranked miR11 and miR624* first followed by miR279d and U6 (Fig 2E). However, the candidate gene miR624* was excluded for stability analysis due to its extreme Ct value (> 35) in all the tissues. Once removed, U6 was considered the most stable gene followed by miR11 (Fig 3).

### Stability of candidate reference genes in tissues of G88-to-Col 0 (GC) strain

**Fig 4:**
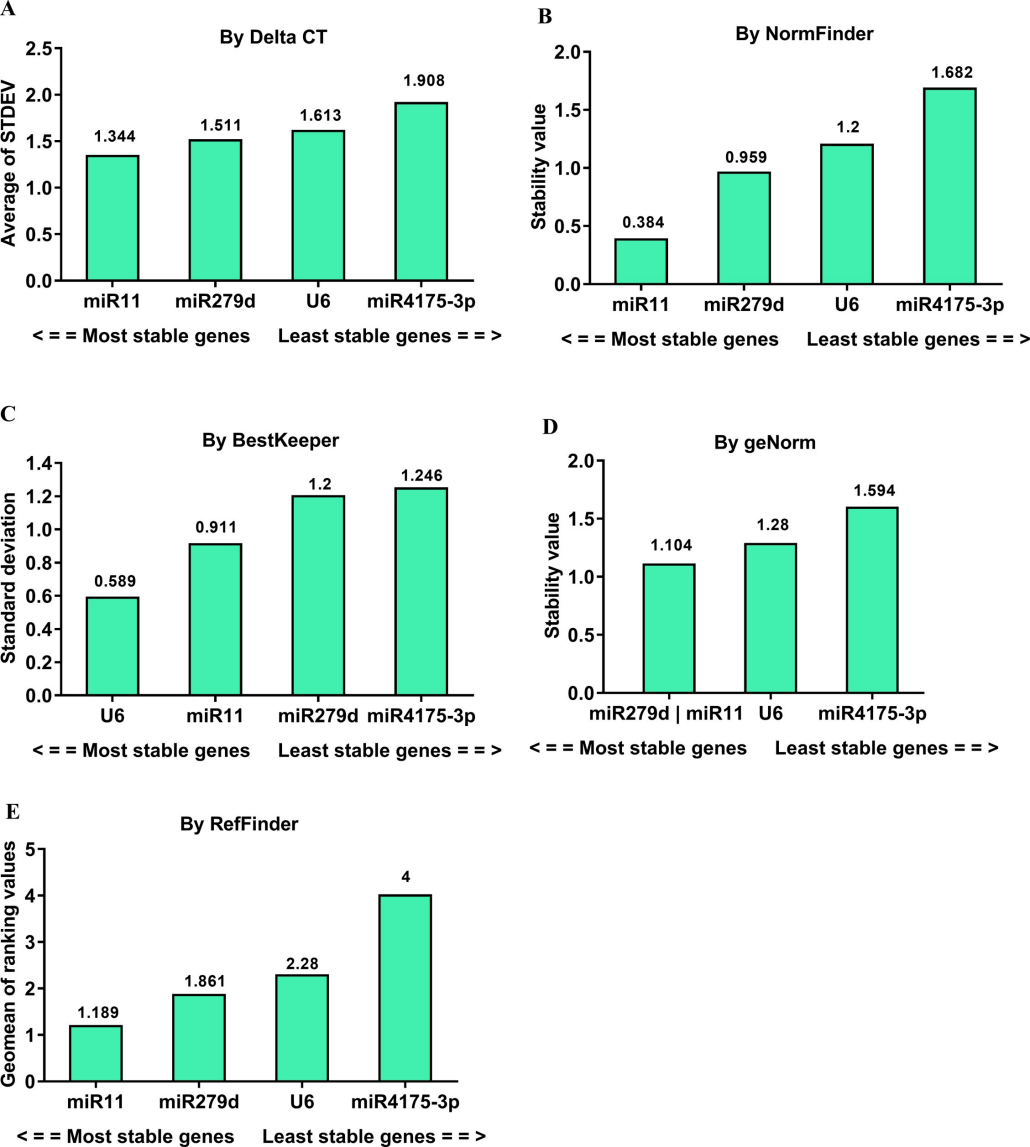
Stability analysis of the candidate reference genes in the tissues of GC strain. The Ct values derived from samples including midgut, silk gland, Malpighian tubule, fat body, hemolymph and remaining tissues from the first-day 4^th^ instar larvae were used to calculate the stability values by different algorithms. A lower value indicates a more stable candidate gene as reference gene (numbers reported above the columns).

To determine whether the stability of candidate genes was altered when the GC strain fed on artificial diet was transferred onto *A*. *thaliana* (producing the GC strain), tissue samples including metabolic and excretory organs and body fluids (hemolymph) dissected from GC strain were used to validate the four reference genes (miR11, miR279d, miR4175-3p and U6). The results showed that the stability ranking differed from that of tissue samples of G88 strain. The gene stability ranked by Delta CT was consistent with NormFinder given that miR11 and miR279d were the most stable reference genes instead of U6 (Fig 4A and 4B), while BestKeeper identified U6 as the most stable reference gene (Fig 4C). Similarly, geNorm identified miR279d and miR11 as the best reference genes (Fig 4D). In both strains, miR11 was identified as one of the most stable genes while miR4175-3p was the least stable (Fig 4E).

### Optimal numbers of reference genes

**Fig 5:**
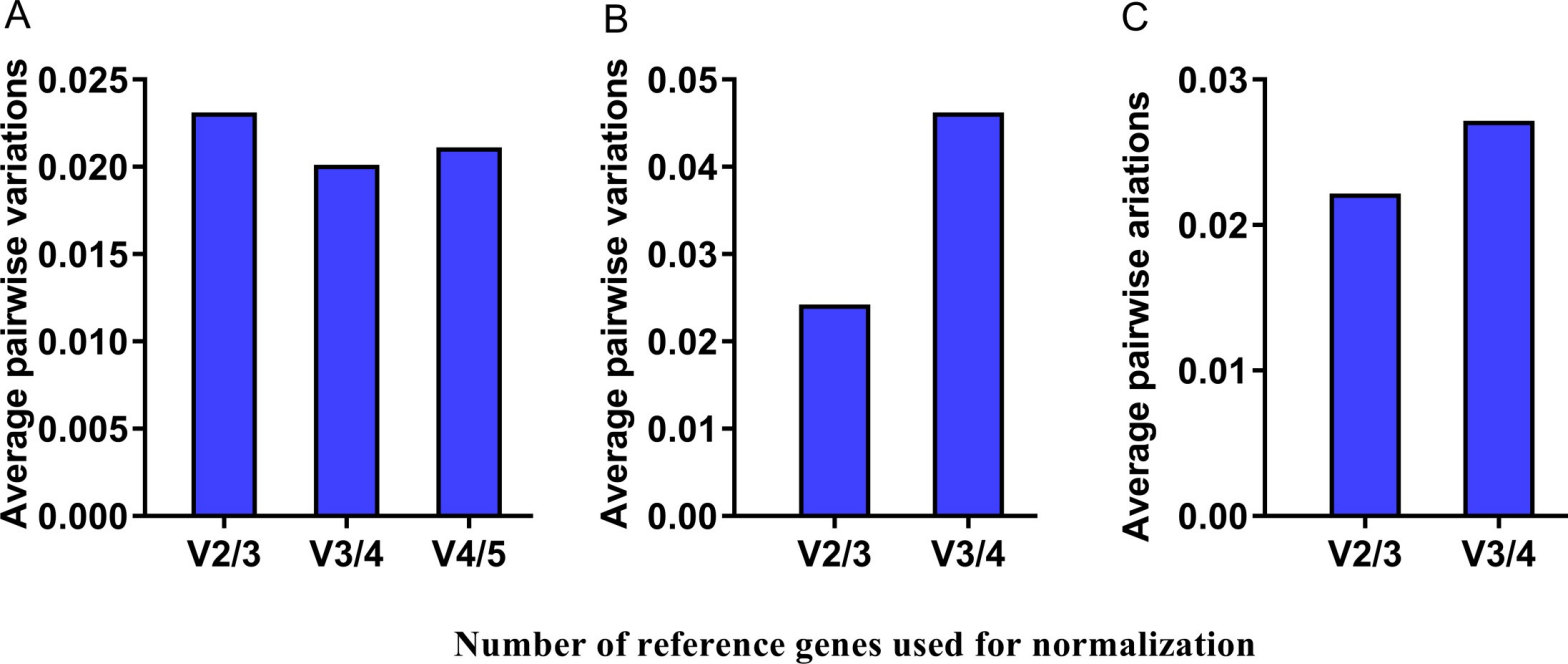
Determination of the optimal number of reference genes for normalization. (A) Developmental stages of G88 strain; (B) Tissues of G88 strain; (C) Tissues of GC strain. Pairwise variations (*V*_*n*/*n+*1_) were calculated between the normalization factors NF_*n*_ and NF_*n*+1_ by geNorm to determine the optimal number of reference genes. geNorm decides whether inclusion of an extra reference gene adds to the stability of the normalization factor.

The program geNorm can be used to determine the optimal number of reference genes for gene expression normalization. Pairwise variation *V*_*n/n+*1_ was calculated between the two sequential normalization factors (NF_*n*_ and NF_*n+*1_), using an average value of pairwise variations (*V* score) less than 0.15 as a guideline [27]. The results showed that all the *V* scores were significantly less than the proposed 0.15 value (Fig 5). For the developmental stages, the three most stable genes provided high quality data (Fig 5A). For both tissue samples of G88 and GC strains, the best two reference genes were sufficiently enough for reliable normalization (Fig 5B and 5C).

### Stability of candidate reference genes measured by ddPCR

To further confirm the stability, the top three reference genes (miR11, miR279d, and U6) selected from tissue samples of the GC strain were subjected to absolute quantification using ddPCR. In the assay for thermal gradient optimization of annealing temperature, the negative and positive droplets were separated from each other and the dispersed droplets were reduced at 55.7–56.9°C as revealed by the 1-D fluorescence amplitude plots (S3–S5 Figs). The optimal annealing temperature was finally set at 56°C. In addition, cDNA of larval midgut of the GC strain was assessed to ensure the concentration loaded within the dynamic range of detection. The appropriate amounts of input DNA were 50 ng, 10 ng and 5 ng for miR11, miR279d and U6, respectively (S3–S5 Figs).

We then detected the absolute expression levels of the top three reference genes (miR11, miR279d and U6) (S6–S8 Figs) and found that miR11 remained as the most stable reference gene based on absolute quantitative levels due to the lowest standard deviation (Table 2).

**Table 2:** Validation of the stability of candidate reference genes using ddPCR.

Gene	n	Mean ± SD	Maximum	Minimum
miR11	6	80.05 ± 24.40	107.00	39.70
miR279d	6	65.24 ± 38.64	135.00	20.90
U6	6	192.96 ± 47.19	275.00	97.20

Data are shown in the form of absolute concentration (copies/μL). The mean, maximum and minimum of absolute concentrations of the sampled tissues of the GC strain are shown. n: number of the tested tissue samples, SD: standard deviation.

### Validation of reference gene selection

**Fig 6:**
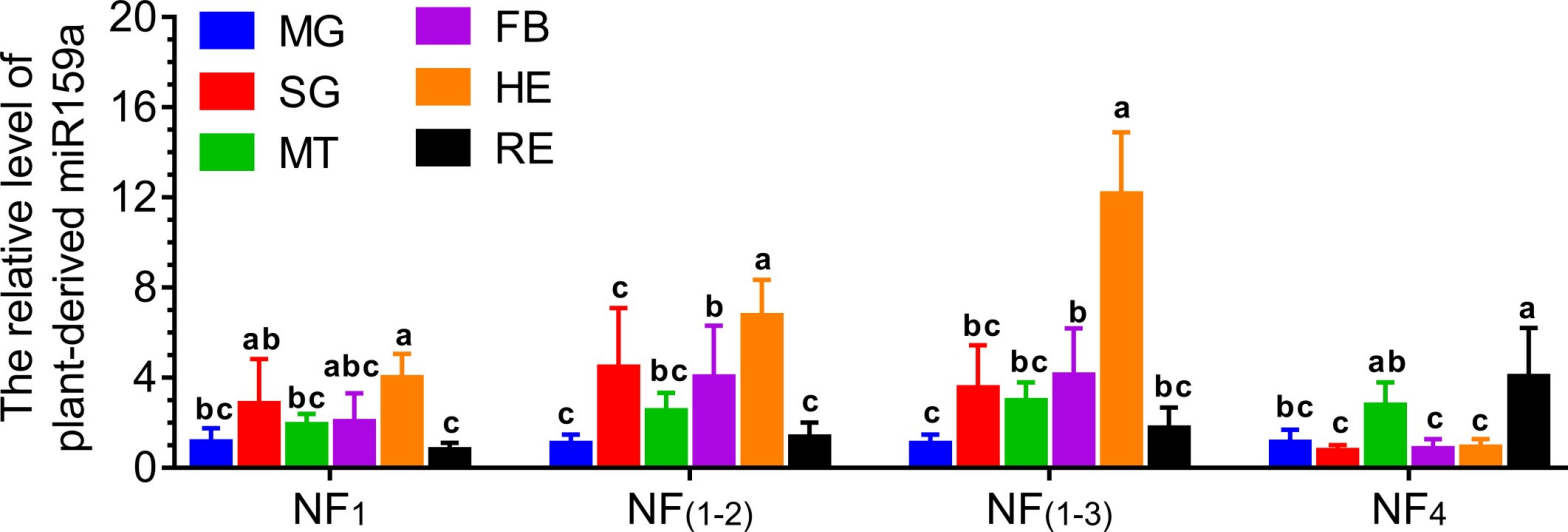
Validation of reference gene selection using qRT-PCR. Relative expression levels of the target gene ath-miR159a in the tissue samples of the GC strain were measured by four normalization factors. NF_1_, normalized against the best reference gene; NF_(1–2)_, normalized against the two most stable reference genes; NF_(1–3)_, normalized against the three most stable reference genes; and NF_4_, normalized against the least stable reference gene. MG: midgut, SG: silk gland, MT: Malpighian tubule, FB: fat body, HE: hemolymph, RE: remaining tissues. Expression levels were independently compared among tissues for each normalization factor. The data were presented as the mean ± SD with level in midgut normalized to 1 (one-way ANOVA followed by a Tukey’s multiple comparison test, *p* < 0.05).

The target miRNA ath-miR159a, a plant-derived miRNA, was only detectable in GC strain samples. Therefore, the levels of ath-miR159a in various tissues of the GC strain were evaluated by using its level in midgut as a control group. The expression patterns were analyzed by four different normalization factors: 1) the best (NF_1_, miR11), 2) the worst (NF_4_, miR4175-3p), and two optimal recommended combinations, 3) NF_(1–2)_, miR11 and miR279d and 4) NF_(1–3)_, miR11, miR279d and U6 (Fig 6). Generally, the expression patterns of ath-miR159a normalized by NF_1_, NF_(1–2)_ and NF_(1–3)_ were similar but distinct from that normalized by NF_4_. The level of ath-miR159a in hemolymph samples was relative high when using NF_1_, NF_(1–2)_ and NF_(1–3)_. However, its level in the remaining tissues became the highest when using NF_4_. There was no difference in ath-miR159a levels between the midgut and the remaining tissues normalized by NF_1_, NF_(1–2)_ and NF_(1–3)_, while a significant difference was found with NF_4_.

**Fig 7:**
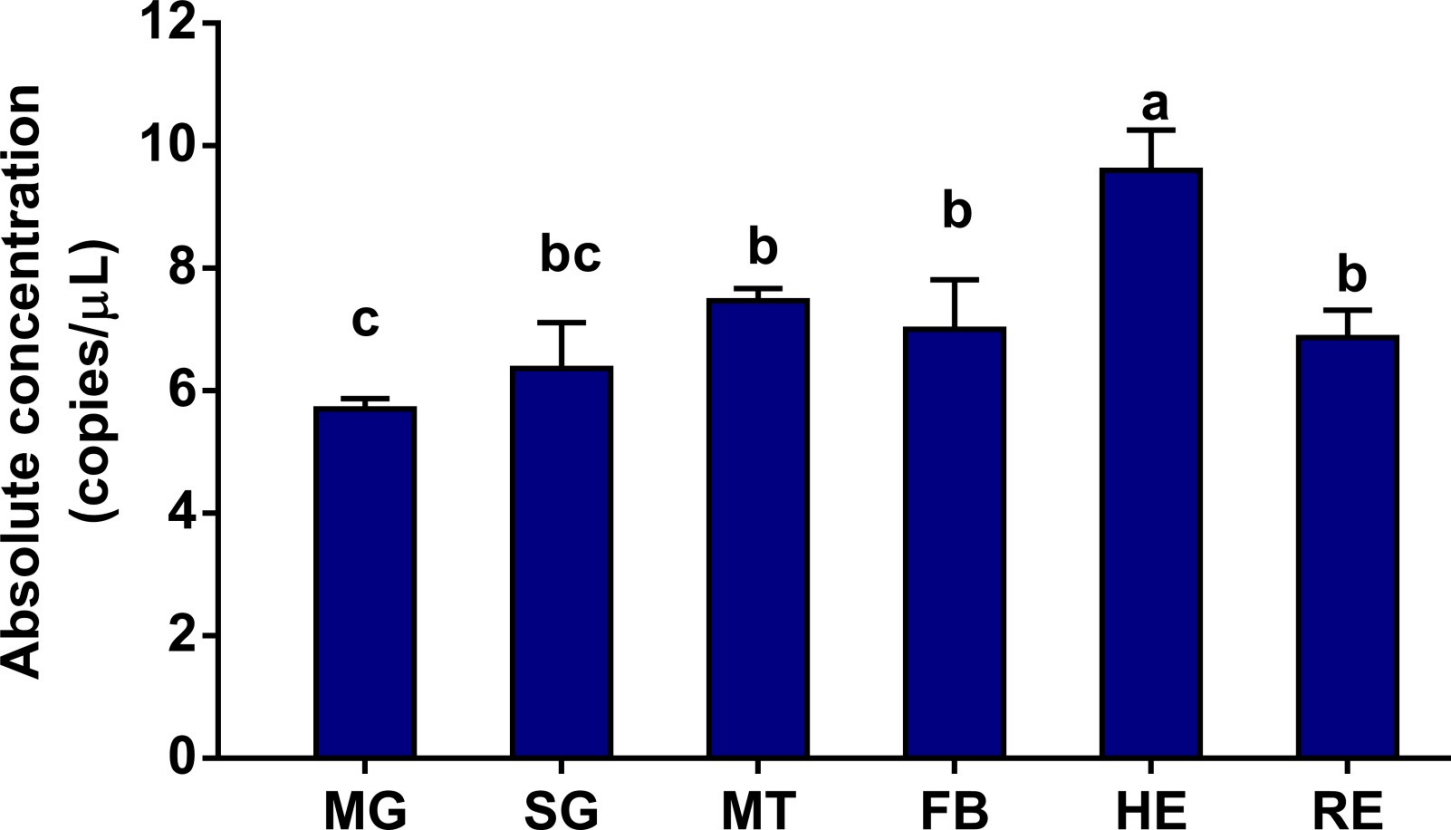
Validation of reference gene selection using ddPCR. MG: midgut, SG: silk gland, MT: Malpighian tubule, FB: fat body, HE: hemolymph, RE: remaining tissues. The data were presented as the mean ± SD (one-way ANOVA followed by a Tukey’s multiple comparison test, *p* < 0.05).

To further evaluate the effects of four normalization factors on the accuracy of the qRT-PCR test, absolute quantification of ath-miR159a was investigated using ddPCR (S9 Fig). Its expression profiling was similar with the expression pattern normalized by NF_1_, NF_(1–2)_ or NF_(1–3)_ in qRT-PCR but varied greatly with NF_4_. We found that ath-miR159a was mostly distributed in hemolymph followed by Malpighian tubule and fat body (Fig 7). Consistent results of qRT-PCR and ddPCR emphasized that miR11 was an ideal reference gene for expression normalization and profiling of plant-derived miRNAs in *P*. *xylostella*.

## Discussion

Detection of expression level of miRNAs using qRT-PCR is fundamental for functional studies in insects [27, 28]. However, the work on selection of reference genes for miRNA quantification in *P*. *xylostella* is limited regarding developmental stages and insecticide resistance [26]. In our study, we built a system that could trace whether plant miRNAs entered the insect body for cross-kingdom gene regulation, by transferring larvae of G88 strain of *P*. *xylostella*, which permanently fed on artificial diet without plant ingredients, onto the host plant *A*. *thaliana*. To avoid nonspecific amplification due to the limit of short miRNA sequences, stem-loop strategy can be used and, cDNA generated using this strategy is exclusive for a single miRNA in every individual sample [29]. Based on this notion, we initially selected seven candidate miRNAs and U6 snRNA. We then assessed the stability of these eight candidate genes over different developmental stages and tissues in the G88 strain before evaluating them in different tissues of the GC strain. We were able to select the most stable reference gene, miR11. Through this study, we also developed a qRT-PCR-based system for the accurate measurement of level of plan-derived miRNAs in *P*. *xylostella*.

Further analyses on Ct values for stability led us to eliminate most of the candidate reference genes. U6 was also a strong candidate but it has been reported that U6 is not always an appropriate reference gene for qRT-PCR. It is likely that the longer RNAs, such as U6, are more easily degraded by RNase after repeated freezing and thawing [7, 8]. In contrast, miRNAs appear to be more stable, particularly those with a high GC ratio or under the protection of protein carrier and exosome [30, 31]. Therefore, it is difficult to identify a universal reference gene for the normalization of gene expression under various circumstances, but gene-specific variation derived from treatment differences in an experiment can be faithfully reflected when using proper reference genes [9, 32]. Previous studies have showed that using multiple reference genes can be more reliable than one single reference gene to capture the variation [33–35]. By comparing the ath-miR159a expression patterns normalized by single or multiple reference gene(s), we were able to select miR11, which produced a similar result to those normalized by reference gene combinations.

Currently, ddPCR is extensively used to detect the number of dietary miRNAs related to cross-kingdom regulation and circulatory miRNAs associated with cancer [18, 36]. With the random partitioning of droplets, ddPCR technology can reliably and precisely detect the slight changes of mRNA or miRNA in a small amount of sample [37]. In our study, we observed that ath-miR159a was much higher in hemolymph, Malpighian tubule, and fat body than in the midgut of the GC strain and was absent in the G88 strain. This finding indicated that the ingested plant miRNAs could break through the insect gut barrier and be absorbed within the whole body of *P*. *xylostella* and may exert some specific functions. The limitations of these results might have been attributed to the fact that target miRNAs were in the lowest amplification length range at 60 bp, which is shorter than recommended amplicon length of 80–250 bp and potentially affected the separation between positive and negative droplets.

Taken together, this study assessed the selected reference genes for quantification of plant-derived miRNAs in *P*. *xylostella* by means of qRT-PCR and ddPCR. After comparing and analyzing the stability of four candidate reference genes, we recommended miR11 to be use as a reference gene. This work was fundamental for revealing cross-kingdom functions of the miRNAs derived from host plant in regulating gene expression of the insect herbivore. These findings may help develop a novel siRNA-based control strategy produced from the plants and free from genetic transformation.

## Materials and methods

### Insects and sampling

The G88 strain of *P*. *xylostella* [38] was introduced and reared on artificial diet under laboratory conditions for more than two years prior to these experiments. Insects were maintained at 25 ± 1°C and a RH of 60 ± 10% with a photoperiod of 16 h: 8 h (L: D). To develop the GC (G88-to-Col 0) strain, newly emerged 1^st^ instar larvae of the G88 strain were transferred on Col 0-type of the host plant *Arabidopsis thaliana* to be reared until the 4^th^ instar.

Individuals of the G88 strain were collected at different developmental stages while only 4^th^ instar larvae of the GC strain were collected. In addition, various tissues of 4^th^ instar larvae of G88 and GC strains and adults of G88 strain were collected. Regarding the developmental stages of G88 strain, each biological replicate contained one of the following to acquire sufficient materials for the analyses: 300 first-day eggs, 50 1^st^ instar larvae, 30 2^nd^ instar larvae, 10 3^rd^ instar larvae, 6 4^th^ instar larvae, 6 pupae or 6 adults. For tissues of G88 strain, the following tissues were collected: brain, midgut, silk gland, Malpighian tubule, fat body, hemolymph, and the remaining tissues of 4^th^ instar larvae (each tissue as a pool of 30 individuals per biological replicate), and adult testes and ovaries (pooled samples of 30 for each biological replicate). For tissues of the GC strain, the same procedure was used to dissect midgut, silk gland, Malpighian tubule, fat body, hemolymph and remaining tissues from the first-day 4^th^ instar larvae for each biological replicate. All specimens or tissues were quickly frozen in liquid nitrogen and stored at -80°C. All following qRT-PCR and ddPCR experiments were based on these three biological replicates.

### Total RNA extraction and cDNA synthesis

Total RNA was extracted using TRIzol kit (Invitrogen, USA) following the manufacturer’s instructions and quantified by a Nanodrop 2000 (Thermo Scientific, USA). RNA samples with a clear 18S rRNA band in the agarose gel electrophoresis and A260/A280 values within 1.9–2.1 were further used for the synthesis of first-strand cDNA of mature miRNA using the GoScript Reverse Transcription System kit (Promega, USA). The gene-specific reverse primer was synthesized by adding the reverse complementary sequence of the last 8 bp of mature miRNA to the 3' terminal of the common stem-loop structure (Table 1). The remainder of the sequence of mature miRNA was added with a universal adapter at the 5' terminal as the forward primer paired with a universal reverse primer. The mature sequences and expression levels of candidate miRNAs from high-throughput sequencing were presented in S2 Table. Tested miRNAs, including a plant-derived miR159a from *A*. *thaliana*, were verified using Sanger sequencing (S10 Fig).

### qRT-PCR assays

qRT-PCR assays were performed using the GoTaq qPCR kit (Promega, USA) and CFX96 Real time PCR system (Bio-rad, USA). The qRT-PCR reaction mixture consisted of 10 μL of 2 × GoTaq qPCR Master Mix, 0.5 μL of 10 μM for each of the gene-specific primer pairs (Table 2), 2 μL of 3 × diluted cDNA sample, and 7 μL of nuclease-free water for a final volume of 20 μL. PCR program was as follows: 95°C for 3 min, 40 cycles of 95°C for 30 s and 58°C for 30 s. At the end of the cycling, the melting curves of the resulting PCR products were obtained by gradually increasing the temperature from 55°C to 95°C at an increment of 0.5°C/5 s to assess the specificity of the primers. Three technical replicates were prepared for the qRT-PCR test of each biological replicate. The candidate reference genes were identified based on the following criteria: the genes were expressed in all samples and the corresponding mean Ct values were ≤ 35.

The PCR efficiency of all the primer pairs was measured using cDNA of 4^th^ instar larvae of the G88 strain. A standard curve for each gene was generated based on the cycle threshold (Ct) values (Y axis) detected from a series of 5-time dilution of cDNA starting from 1 μg (X axis, log_5_ transformation) to establish the standard curve. The regression equation was developed to calculate the slope and regression coefficient (*R*^2^) of each primer pair. The corresponding qRT-PCR efficiency (*E*) was calculated based on the equation: (10^[-1/slope]^-1) × 100%. The results were typically acceptable when R^2^ > 0.98 with a PCR efficiency between 90% and 110% [39].

### ddPCR procedure

The samples used for qRT-PCR were further validated using ddPCR. Each ddPCR assay was loaded into a disposable droplet generator cartridge, containing 10 μL of QX200 EvaGreen ddPCR Supermix, 1 μL of 10—fold or 50—fold diluted cDNA, 1 μL of the recommended optimal concentration 100 nM for each of the gene-specific primer pairs and nuclease-free water up to 22 μL. The QX200 droplet generator was used to partition the reaction mixture into aqueous droplets in oil solution. Droplets were then transferred onto a 96-well PCR plate and subsequently heat-sealed with foil. Thermal cycling was performed using the C1000 Touch Thermal Cycler (Bio-rad, USA) with the following procedure: 95°Cfor 5 min, 40 cycles of 95°C for 30 s and 58°C for 1 min, a signal stabilization step at 4°C for 5 min, 90°C for 5 min and infinite holding at 4°C. The temperature ramp rate was set to 2°C/s for each step during the cycling. A no template control was included in every assay. After PCR, the wells containing the droplets were analyzed by QX200 Droplet Reader (Bio-rad, USA).

To obtain the largest fluorescence amplitude difference between the positives and negatives and avoid nonspecific amplification, thermal gradient optimization of ddPCR assay was performed in the C1000 Touch Thermal Cycler by replacing the annealing temperature of the standard PCR cycling program with a thermal gradient from 55°C to 65°C. Eight ddPCR reactions containing the same amount of cDNA were annealed at different annealing temperatures. Three loaded cDNAs of the larval midgut of the GC strain, including 5 ng, 10 ng, and 50 ng, were subjected to thermal gradient optimization for annealing temperature.

### Validation of reference gene selection

The miR159a of *A*. *thaliana* (ath-miR159a), a cross-kingdom miRNA, was used to assess the validity of selected reference genes. The levels of ath-miR159a were determined in different tissues of GC strain using qRT-PCR and were normalized by the Ct value of the best normalization factor NF_1_ or the worst NF_4,_ or geometric mean Ct value of the optimal recommended combination of NF_(1–2)_ or NF_(1–3)_. The relative expression levels were calculated using the 2^-ΔΔCt^ method. The expression pattern of ath-miR159a was also measured in different tissues of the GC strain using ddPCR.

### Statistical analysis

The comprehensive ranking of candidate reference genes was assessed by a web-based analysis tool RefFinder [40], which contains four algorithms: Delta CT [41], BestKeeper [42], geNorm [43] and NormFinder [44]. Raw Ct values were loaded directly for analysis using BestKeeper. The Ct values were transformed to linear scale expression values before analysis by Delta CT, geNorm and NormFinder algorithms. The stability of the reference genes was evaluated through the standard deviation of Delta CT (SD) and BestKeeper (SD) and the stability values of geNorm (M) and NormFinder (SV). Based on the ranking from each program, RefFinder assigns an appropriate weight to each gene and calculates the geometric mean of their weights for the overall final ranking [41]. For ddPCR, automatic analysis based on a Poisson distribution was applied for distinguishing positive and negative droplets or a threshold was manually set when a concentration estimate fails to appear. Data were analyzed using one-way ANOVA followed by a Tukey’s multiple comparison test performed in IBM SPSS Statistic 21 (IBM, USA). The difference was considered statistically significant at *p* < 0.05.

## Supporting information

S1 Table Cycle threshold (Ct) values of candidate reference genes and target miRNA in all samples. (XLS)

S2 Table Candidate miRNAs selected from small RNA sequencing. (XLS)

S1 Fig Standard curve for each reference gene. (A) miR279d. (B) miR11. (C) miR3281. (D miR624*. (E) miR4175-3p. (F) U6.
(PDF)

S2 Fig Melting curve for each reference gene. (A) miR279d. (B) miR11. (C) miR3281. (D) miR624*. (E) miR4175-3p. (F) U6.
(PDF)

S3 Fig 1**-D fluorescence amplitude plots (left) and histograms (right) for miR11.** For the plots, blue dots denote the positive droplets and gray dots denote the negative droplets. Temperature gradients are from 65°C to 55°C in the order of columns A to H, including 65°C, 64.3°C, 63°C, 61.1°C, 58.8°C, 56.9°C, 55.7°C and 55°C. For the histograms, the left peak represents the frequency of negative droplets and the right peak represents positive droplets. Concentrations of loaded cDNAs are 50 ng, 10 ng and 5 ng for panels (AA'), (BB') and (CC'), respectively.
(PDF)

S4 Fig **1-D fluorescence amplitude plots (left) and histograms (right) for miR279d.** For the plots, blue dots denote the positive droplets and gray dots denote the negative droplets. Temperature gradients are from 65°C to 55°C in the order of columns A to H, including 65°C, 64.3°C, 63°C, 61.1°C, 58.8°C, 56.9°C, 55.7°C and 55°C. For the histograms, the left peak represents the frequency of negative droplets and the right peak represents positive droplets. Concentrations of loaded cDNAs are 50 ng, 10 ng and 5 ng for panels (AA'), (BB') and (CC'), respectively.
(PDF)

S5 Fig **1-D fluorescence amplitude plots (left) and histograms (right) for U6.** For the plots, blue dots denote the positive droplets and gray dots denote the negative droplets. Temperature gradients are from 65°C to 55°C in the order of columns A to H, including 65°C, 64.3°C, 63°C, 61.1°C, 58.8°C, 56.9°C, 55.7°C and 55°C. For the histograms, the left peak represents the frequency of negative droplets and the right peak represents positive droplets. Concentrations of loaded cDNAs are 50 ng, 10 ng and 5 ng for panels (AA'), (BB') and (CC'), respectively.
(PDF)

S6 Fig **1-D fluorescence amplitude plots (left) and histograms (right) for absolute quantification of miR11 in tissues of GC strain.** The ddPCR assays are performed in three biological replicates (AA', BB' and CC'). For the plots, blue dots denote the positive droplets and gray dots denote the negative droplets. Columns A to G represent tissue samples of GC strain in the order of midgut, silk gland, Malpighian tubule, fat body, hemolymph and remaining tissues. Column H is the no template control for A and B, and pure water control for C with no droplets amplified. For the histograms, the left peak represents the frequency of negative droplets and the right peak represents positive droplets.
(PDF)

S7 Fig **1-D fluorescence amplitude plots (left) and histograms (right) for absolute quantification of miR279d in tissues of GC strain.** The ddPCR assays are performed in three biological replicates (AA', BB' and CC'). For the plots, blue dots denote the positive droplets and gray dots denote the negative droplets. Columns A to G represent tissue samples of GC strain in the order of midgut, silk gland, Malpighian tubule, fat body, hemolymph and remaining tissues. Column H is the no template control (NTC). For the histograms, the left peak with amplitudes from 0 to 2000 represents the frequency of NTC. The middle peak with amplitudes approximately 6000 is the frequency of negative droplets and the remainder represents positive droplets, which were reduced in the tested samples, leading to indistinctive peaks.
(PDF)

S8 Fig **1-D fluorescence amplitude plots (left) and histograms (right) for absolute quantification of U6 in tissues of GC strain.** The ddPCR assays are performed in three biological replicates (AA', BB' and CC'). For the plots, blue dots denote the positive droplets and gray dots denote the negative droplets. Columns A to G represent tissue samples of GC strain in the order of midgut, silk gland, Malpighian tubule, fat body, hemolymph and remaining tissues. Column H is the no template control for A and B, and pure water control for C with no droplets amplified. For the histograms, the left peak represents the frequency of negative droplets and the right peak represents positive droplets.
(PDF)

S9 Fig **1-D fluorescence amplitude plots (left) and histograms (right) for absolute quantification of ath-miR159a in tissues of GC strain.** The ddPCR assays are performed in three biological replicates (AA', BB' and CC'). For the plots, blue dots denote the positive droplets and gray dots denote the negative droplets. Columns A to G represent tissue samples of GC strain in the order of midgut, silk gland, Malpighian tubule, fat body, hemolymph and remaining tissues. Column H is the no template control (NTC). For the histograms, the left peak with amplitudes from 0 to 5000 represents the frequency of NTC, the middle peak with amplitudes approximately 10000 is the frequency of negative droplets, and the remainder represent positive droplets, which were reduced in the tested samples leading to indistinctive peaks.
(PDF)

S10 Fig Sanger sequencing of seven candidate miRNAs selected from small RNA sequencing and the target miRNA (ath-miR159a). (PDF)
