# Supplementary figures and images for: Selection of reference genes for expression analysis of plant-derived microRNAs in Plutella xylostella using qRT-PCR and ddPCR
Source: PLoS One. 2019 Aug 1;14(8):e0220475. doi: 10.1371/journal.pone.0220475 (PMC6675394; doi:10.1371/journal.pone.0220475)

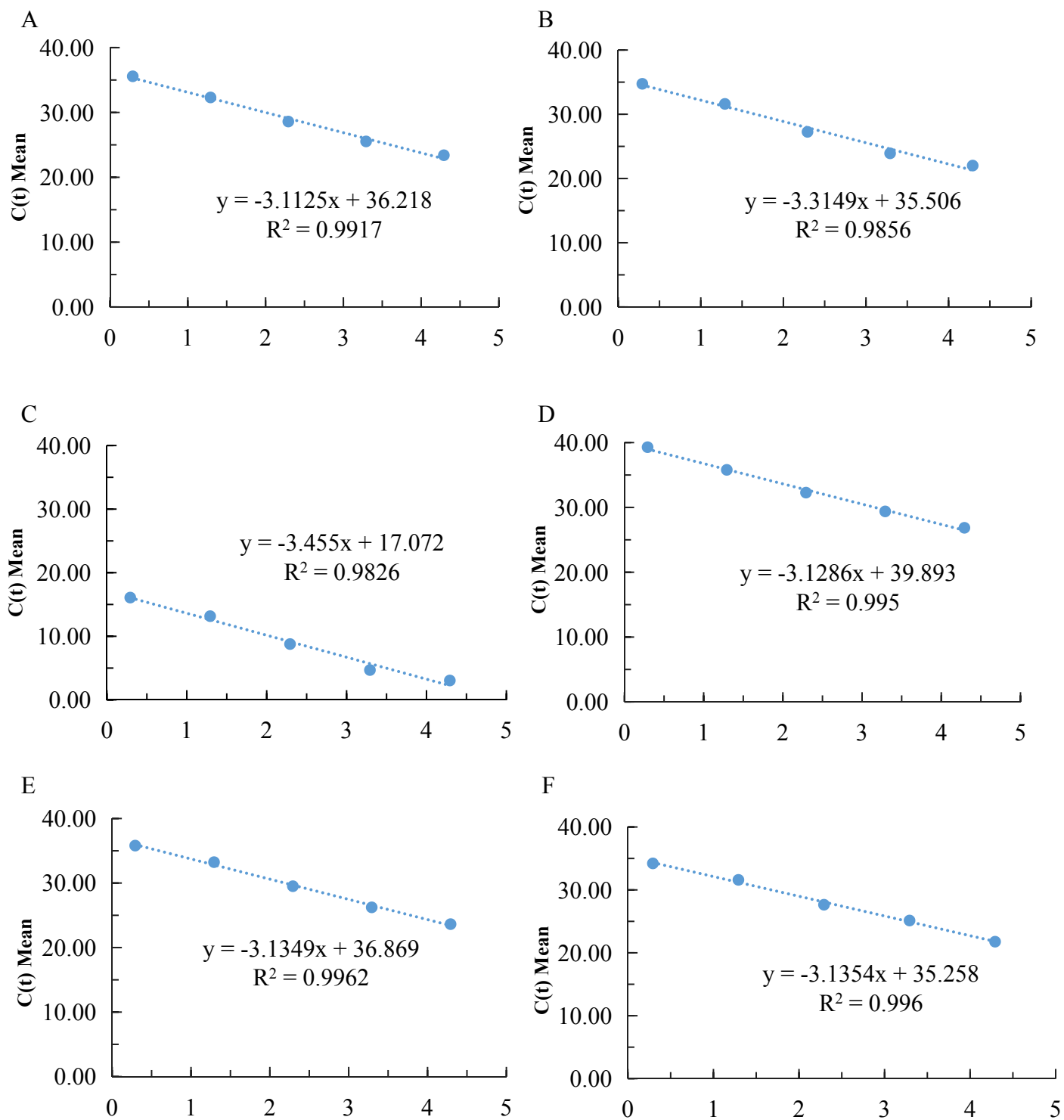

**S1 Fig. Standard curve for each reference gene.** (A) miR279d. (B) miR11. (C) miR3281. (D) miR624\*. (E) miR4175-3p. (F) U6.

Supplement: S1 Fig — (A) miR279d. (B) miR11. (C) miR3281. (D miR624*. (E) miR4175-3p. (F) U6. (PDF) [file pone.0220475.s003.pdf]

A

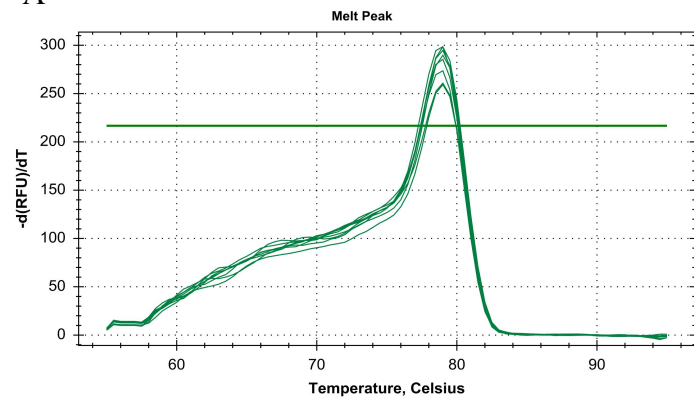

B

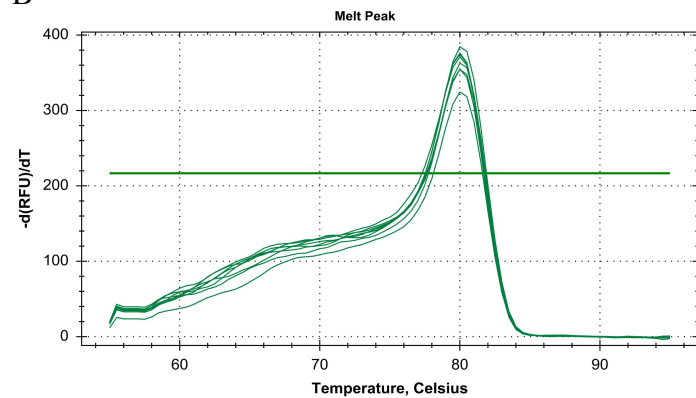

C

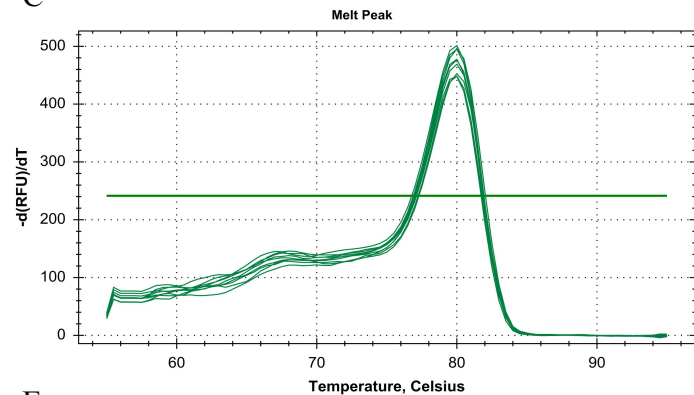

D

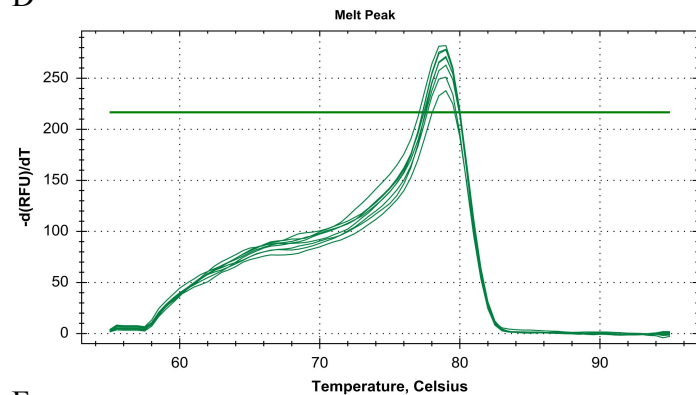

E

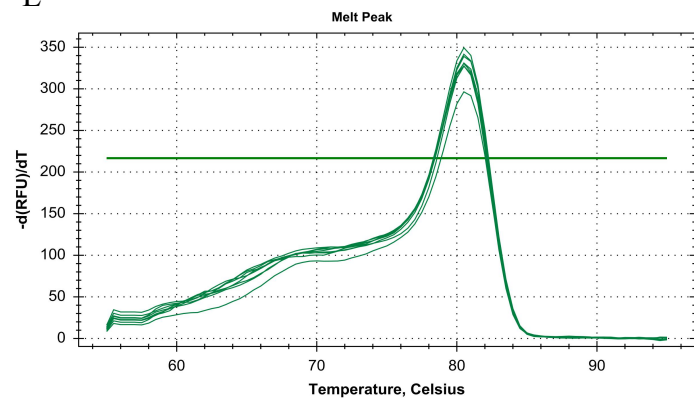

F

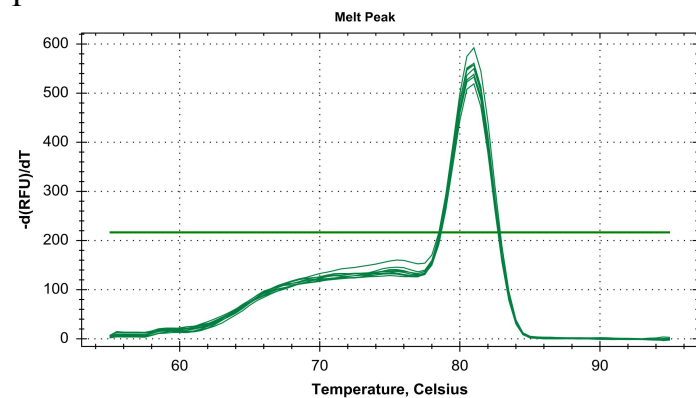

**S2 Fig. Melting curve for each reference gene.** (A) miR279d. (B) miR11. (C) miR3281. (D) miR624\*. (E) miR4175-3p. (F) U6.

Supplement: S2 Fig — (A) miR279d. (B) miR11. (C) miR3281. (D) miR624*. (E) miR4175-3p. (F) U6. (PDF) [file pone.0220475.s004.pdf]
