# Supplementary material for: Selection of reference genes for expression analysis of plant-derived microRNAs in Plutella xylostella using qRT-PCR and ddPCR
Source: PLoS One. 2019 Aug 1;14(8):e0220475. doi: 10.1371/journal.pone.0220475 (PMC6675394; doi:10.1371/journal.pone.0220475)
Supplement: S3 Fig — 1-D fluorescence amplitude plots (left) and histograms (right) for miR11. For the plots, blue dots denote the positive droplets and gray dots denote the negative droplets. Temperature gradients are from 65°C to 55°C in the order of columns A to H, including 65°C, 64.3°C, 63°C, 61.1°C, 58.8°C, 56.9°C, 55.7°C and 55°C. For the histograms, the left peak represents the frequency of negative droplets and the right peak represents positive droplets. Concentrations of loaded cDNAs are 50 ng, 10 ng and 5 ng for panels (AA'), (BB') and (CC'), respectively. (PDF) [file pone.0220475.s005.pdf]

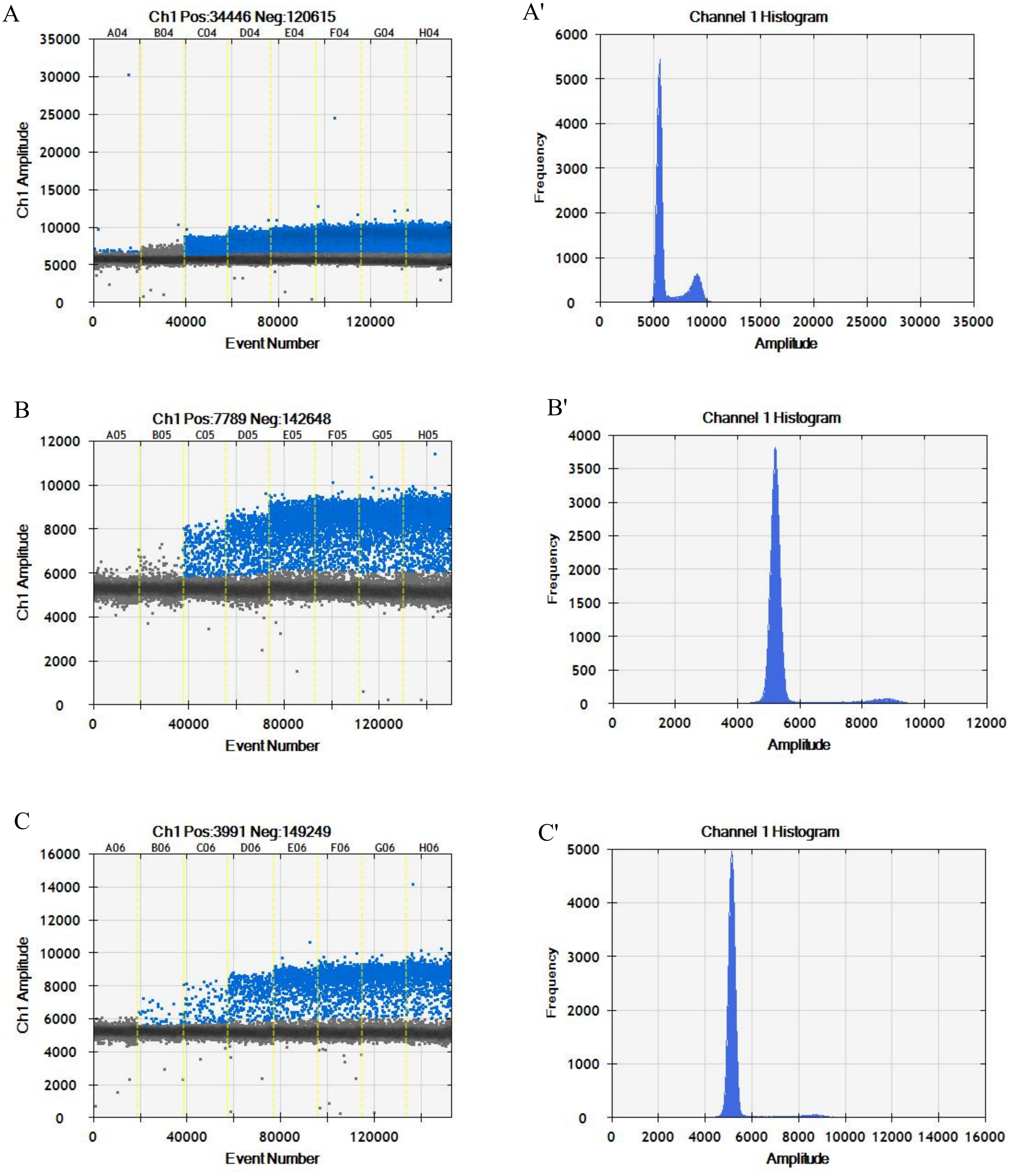

S3 Fig. 1-D fluorescence amplitude plots (left) and histograms (right) for miR11. For the plots, blue dots denote the positive droplets and gray dots denote the negative droplets. Temperature gradients are from 65 ℃ to 55 ℃ in the order of columns A to H, including 65 ℃, 64.3 ℃, 63 ℃, 61.1 ℃, 58.8 ℃, 56.9 ℃, 55.7 ℃ and 55 ℃. For the histograms, the left peak represents the frequency of negative droplets and the right peak represents positive droplets. Concentrations of loaded cDNAs are 50 ng, 10 ng and 5 ng for panels (AA'), (BB') and (CC'), respectively.
