# Supplementary material for: Selection of reference genes for expression analysis of plant-derived microRNAs in Plutella xylostella using qRT-PCR and ddPCR
Source: PLoS One. 2019 Aug 1;14(8):e0220475. doi: 10.1371/journal.pone.0220475 (PMC6675394; doi:10.1371/journal.pone.0220475)
Supplement: S8 Fig — 1-D fluorescence amplitude plots (left) and histograms (right) for absolute quantification of U6 in tissues of GC strain. The ddPCR assays are performed in three biological replicates (AA', BB' and CC'). For the plots, blue dots denote the positive droplets and gray dots denote the negative droplets. Columns A to G represent tissue samples of GC strain in the order of midgut, silk gland, Malpighian tubule, fat body, hemolymph and remaining tissues. Column H is the no template control for A and B, and pure water control for C with no droplets amplified. For the histograms, the left peak represents the frequency of negative droplets and the right peak represents positive droplets. (PDF) [file pone.0220475.s010.pdf]

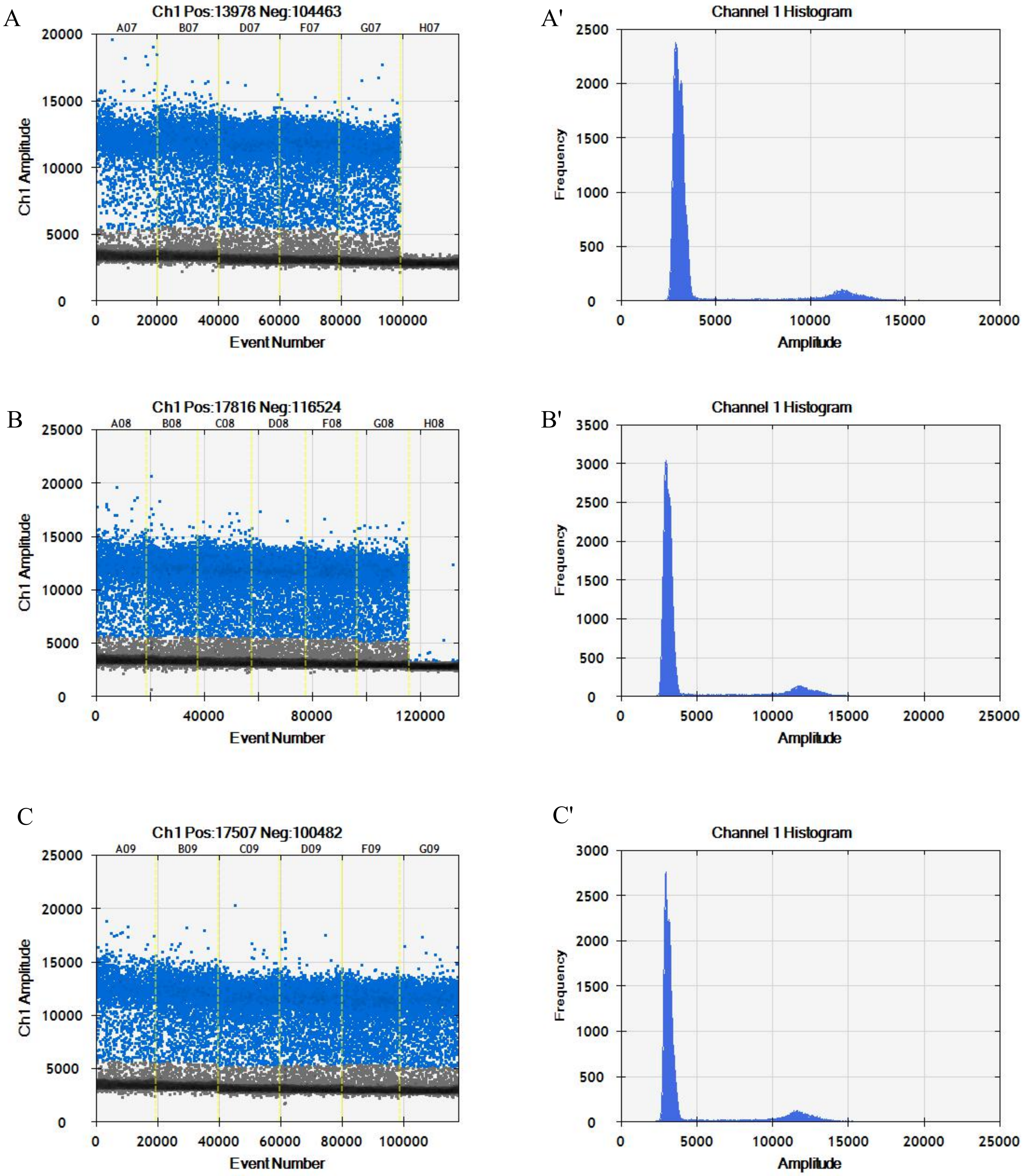

S8 Fig. 1-D fluorescence amplitude plots (left) and histograms (right) for absolute quantification of U6 in tissues of GC strain. The ddPCR assays are performed in three biological replicates (AA', BB' and CC'). For the plots, blue dots denote the positive droplets and gray dots denote the negative droplets. Columns A to G represent tissue samples of GC strain in the order of midgut, silk gland, Malpighian tubule, fat body, hemolymph and remaining tissues. Column H is the no template control for A and B, and pure water control for C with no droplets amplified. For the histograms, the left peak represents the frequency of negative droplets and the right peak represents positive droplets.
