# Supplementary material for: Selection of reference genes for expression analysis of plant-derived microRNAs in Plutella xylostella using qRT-PCR and ddPCR
Source: PLoS One. 2019 Aug 1;14(8):e0220475. doi: 10.1371/journal.pone.0220475 (PMC6675394; doi:10.1371/journal.pone.0220475)
Supplement: S9 Fig — 1-D fluorescence amplitude plots (left) and histograms (right) for absolute quantification of ath-miR159a in tissues of GC strain. The ddPCR assays are performed in three biological replicates (AA', BB' and CC'). For the plots, blue dots denote the positive droplets and gray dots denote the negative droplets. Columns A to G represent tissue samples of GC strain in the order of midgut, silk gland, Malpighian tubule, fat body, hemolymph and remaining tissues. Column H is the no template control (NTC). For the histograms, the left peak with amplitudes from 0 to 5000 represents the frequency of NTC, the middle peak with amplitudes approximately 10000 is the frequency of negative droplets, and the remainder represent positive droplets, which were reduced in the tested samples leading to indistinctive peaks. (PDF) [file pone.0220475.s011.pdf]

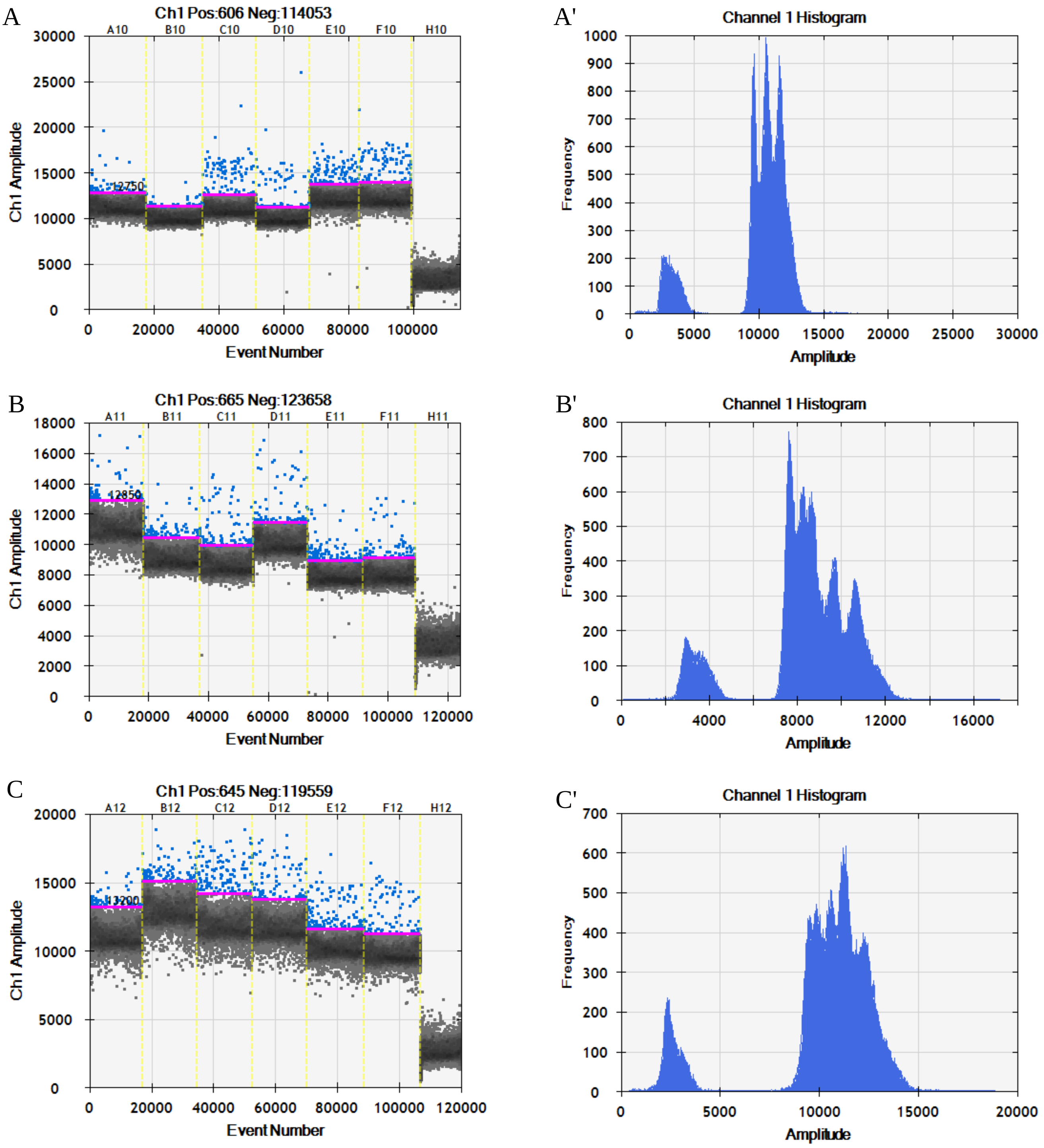

S9 Fig. 1-D fluorescence amplitude plots (left) and histograms (right) for absolute quantification of ath-miR159a in tissues of GC strain. The ddPCR assays are performed in three biological replicates (AA', BB' and CC'). For the plots, blue dots denote the positive droplets and gray dots denote the negative droplets. Columns A to G represent tissue samples of GC strain in the order of midgut, silk gland, Malpighian tubule, fat body, hemolymph and remaining tissues. Column H is the no template control (NTC). For the histograms, the left peak with amplitudes from 0 to 5000 represents the frequency of NTC, the middle peak with amplitudes approximately 10000 is the frequency of negative droplets, and the rest remainder represent positive droplets, which were reduced in the tested samples leading to indistinctive peaks.
