# Supplementary material for: Selection of reference genes for expression analysis of plant-derived microRNAs in Plutella xylostella using qRT-PCR and ddPCR
Source: PLoS One. 2019 Aug 1;14(8):e0220475. doi: 10.1371/journal.pone.0220475 (PMC6675394; doi:10.1371/journal.pone.0220475)
Supplement: S10 Fig — (PDF) [file pone.0220475.s012.pdf]

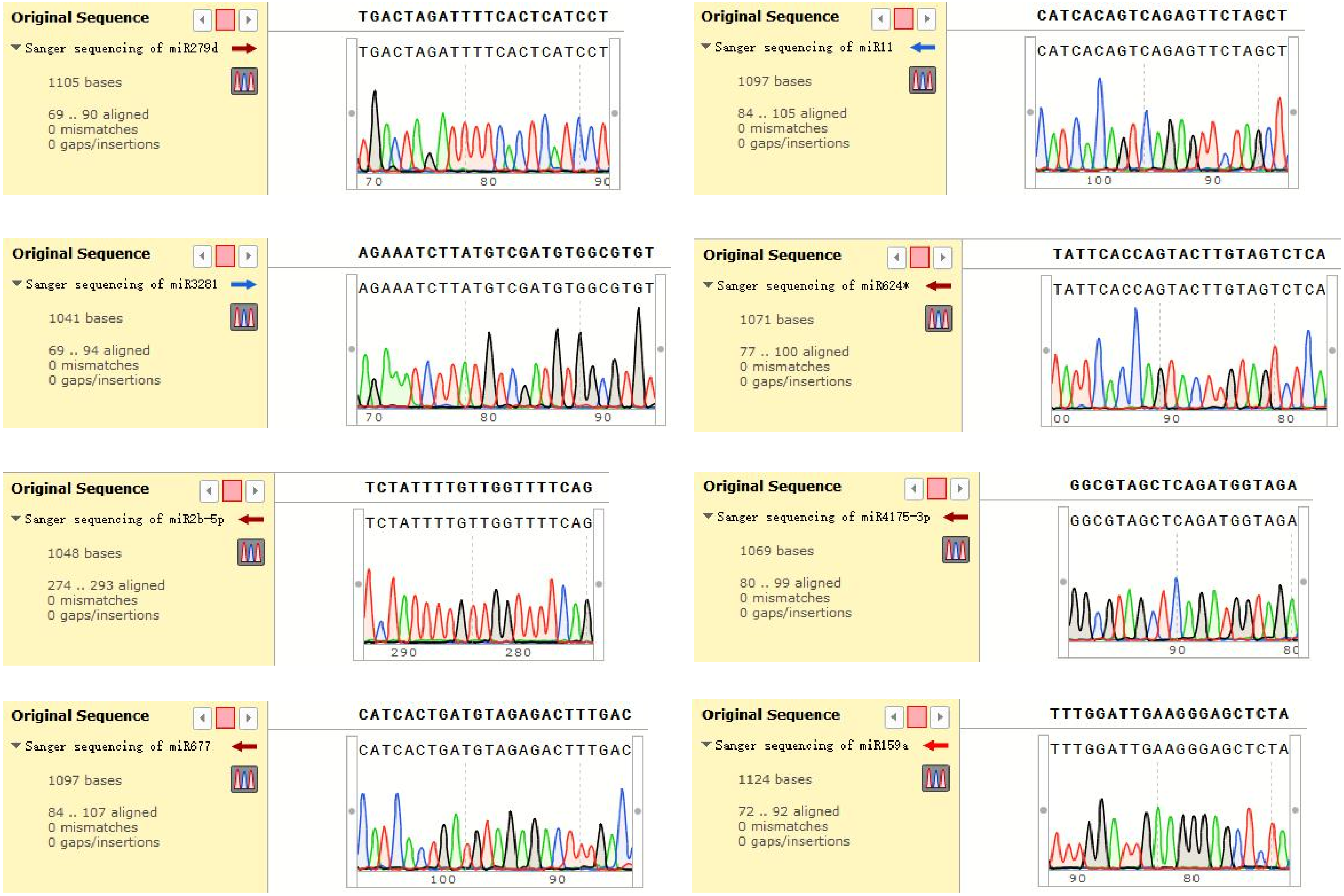

S10 Fig. Sanger sequencing of seven candidate miRNAs selected from small RNA sequencing and the target miRNA (ath-miR159a).
